# Epidemic Tetragenaemia

**Published:** 1916-03

**Authors:** 


**Epidemic Tetragenaemia.** Fernand Tremolieres and Pierre Loew, Paris Med. Nos. 5 and 9, 1915, state that this infection is common in the present war and describe three types: benign, grave and pleuro-pulmonic.

